# New Appliances and Things Medical

**Published:** 1899-10-14

**Authors:** 


### NEW APPLIANCES AND THINGS MEDICAL.

**[We shall be glad to receive, at our Office, 28 & 29, Southampton Street, Strand, London, W.C., from the manufacturers, specimens of all new preparations and appliances which may be brought out from time to time.]**

### SOUTH AUSTRALIAN BRANDY, "ORION" BRAND.

(South Australian Government Bonded Depôt, 35, Walbrook.)

The "Orion" brandy is made under the supervision of the Government excise officers of South Australia, and is certified by H.M. Customs of the colony to be a pure grape spirit and free from adulteration. We have ourselves examined this brandy, and find it a pleasant spirit, clean and wholesome, and very serviceable for invalid use. Alcohol, the result of fermentative change in the starch or sugar of grain, beetroot, and molasses, has undoubtedly a stimulating action on the heart and circulation generally, but the vast superiority of alcohol obtained from vinous sources, and due to the fermentation of the juice of the grape, is perhaps the most definitely proved fact in clinical experience. The therapeutic efficiency of a genuine brandy is undoubtedly due to the various volatile and aromatic ethers which age and natural oxidation processes impart to the distillation product of the fermented juice of the grape. Technical knowledge and recent advances in laboratory methods have enabled the manufacturers of spurious brandies to impart to indifferent spirit, distilled from diverse sources, a superficial similarity to genuine cognac; the alcoholic properties *per se* are, of course, present, but the valued effects of the ethers are absent, or at a minimum. The "Orion" brandy can be relied on as a pure grape spirit, containing pleasant and natural ethers. As time and experience advance, doubtless the natural facilities of South Australia will lead to the production of a brandy quite equal in flavour to the very best cognac from the Charente district in France.

